# Role and Clinical Utility of Cancer/Testis Antigens in Head and Neck Squamous Cell Carcinoma

**DOI:** 10.3390/cancers13225690

**Published:** 2021-11-14

**Authors:** Sharon Changshan Wu, Karl Münger

**Affiliations:** 1 Molecular Microbiology Program, Graduate School of Biomedical Sciences, Tufts University School of Medicine, Boston, MA 02111, USA; Sharon_c.wu@tufts.edu; 2 Department of Developmental, Molecular, and Chemical Biology, Tufts University School of Medicine, Boston, MA 02111, USA

**Keywords:** CT antigens, HNSCC, HPV

## Abstract

**Simple Summary:**

In this review, we discuss the roles of cancer/testis antigens in the germline and their contributions to oncogenic cellular processes. Specifically, we focus on their clinical utility in head and neck squamous cell carcinoma and consider how cancer/testis antigens differentially expressed in HPV-positive HNSCC might contribute mechanistically to the genesis and clinical characteristics of these cancers.

**Abstract:**

Cancer/testis (CT) antigens exhibit selective expression predominantly in immunoprivileged tissues in non-pathological contexts but are aberrantly expressed in diverse cancers. Due to their expression pattern, they have historically been attractive targets for immunotherapies. A growing number of studies implicate CT antigens in almost all hallmarks of cancer, suggesting that they may act as cancer drivers. CT antigens are expressed in head and neck squamous cell carcinomas. However, their role in the pathogenesis of these cancers remains poorly studied. Given that CT antigens hold intriguing potential as therapeutic targets and as biomarkers for prognosis and that they can provide novel insights into oncogenic mechanisms, their further study in the context of head and squamous cell carcinoma is warranted.

## 1. Introduction

Head and neck squamous cell carcinoma (HNSCC), comprising cancers derived from the oral cavity, nasopharynx, oropharynx, hypopharynx, and larynx, accounted for 796,577 new cancer cases and 387,117 cancer-related deaths in 2020 worldwide [1,2]. While around one-third of patients present with early-stage disease and have a favorable prognosis with surgery or radiotherapy, the 5-year overall survival for advanced-stage disease is ~50% and recurrent or metastatic disease is largely incurable [3,4,5]. Thus, there remains a pressing clinical need to improve the therapeutic arsenal against this disease. Major risk factors include tobacco usage, excess alcohol consumption, environmental pollution, and infections with oncogenic human papillomaviruses (HPVs) [1]. Clinically, HPV-positive HNSCCs exhibit a distinct clinical course compared to HPV-negative disease. In particular, patients with HPV-associated oropharyngeal cancers have improved prognosis compared to those with HPV-negative cancers due in part to enhanced responsiveness to chemotherapy and radiotherapy [4]. However, treatment-related morbidities and quality of life issues remain major concerns for survivors [1]. Long-term morbidities include dysphagia, xerostomia, ototoxicity, and trismus [6]. Head and neck cancer survivors have the second-highest suicide rate, surpassed only by pancreatic cancer, and are almost twice as likely to die from suicide compared to survivors of other cancers [7]. Given the motivation to alleviate treatment-associated morbidity and the recognition that a subset of HPV-positive HNSCC patients has a poor prognosis, there is a need to identify biomarkers that define patient subgroups by prognosis or likelihood of safe response to treatment de-escalation [4,8]. Furthermore, why most HPV-positive HNSCCs have a better prognosis compared to HPV-negative is a question of interest in the field. Finally, HNSCC suffers from a dearth of approved targeted therapies [5]. Cancer/testis antigens represent a compelling class of molecules that may fill a niche in each of these research needs. 

## 2. Cancer-Testis Antigens

### 2.1. Overview

Cancer-testis (CT) antigens encompass a set of proteins whose expression is predominantly limited to germ cells or trophoblasts but is aberrantly activated in cancers [9,10,11]. Due to their immunogenicity and limited expression, CT antigens have sparked interest as attractive targets for tumor-specific immunotherapies. The first CT antigen, melanoma antigen (MAGE)-1—now known as MAGEA1—was discovered in 1991 using autologous typing, a technique in which tumor cells from a patient are co-cultured with autologous lymphocytes to test for the generation of T cells that target tumor antigens [10,12,13]. Autologous cytotoxic T lymphocytes targeting melanoma cells from a patient with a favorable clinical course were generated and the gene encoding one of the targeted tumor antigens was cloned and named MAGE1 [10,12]. MAGE2 and MAGE3 were also identified in the study, revealing MAGE to be a gene family [12]. Other CT antigens including B melanoma antigen (BAGE) [14] and G antigen 1 (GAGE1) [15] were subsequently discovered using cytotoxic T lymphocytes from the same patient [10]. Serological analysis of cDNA expression libraries (SEREX), in which cDNA expression libraries are screened using patient antibodies, was the subsequent dominant approach for CT antigen discovery and led to the identification of SSX [16], synaptonemal complex protein 1 (SYCP1) [17], and the New York esophageal squamous cell carcinoma 1 (NY-ESO-1) [18], among others [10]. Approaches to identify novel CT antigens have expanded to in silico identification based on mRNA expression patterns [9,11,19]. While this shift has augmented the CT antigen cohort, a caveat is that the coding products of some of these CT genes have not been formally tested for their immunogenicity [9]. The Cancer-Testis database (CTdatabase), a list of CT genes compiled based on the literature and computational prediction, currently contains 276 genes [13]. A larger list of 1019 CT genes has been compiled through transcriptomic analysis integrating data from multiple publicly available datasets across normal tissues and 19 cancer types [19]. The abundance of available expression data has revealed the expression of genes previously characterized as CT-restricted in other normal tissues. Thus, the description of CT antigens has evolved from “testis-restricted” to the less stringent “testis-preferred”, with further sub-classification into “testis-restricted”, “testis-brain restricted”, and “testis-selective” groups [11].

A separate classification for CT genes can be made based on chromosomal location: CT-X genes encoded on the X chromosome and non-X CT genes encoded autosomally [10]. CT-X genes comprise several multi-gene families, including MAGE, GAGE, PAGE, SSX, CTAG, and SPANX [9,20]. CT-X genes are largely expressed in spermatogonia while non-X CT genes seem to be preferentially expressed in later stages of germ cell differentiation [10].

### 2.2. CT Antigens in Immunotherapy

CT antigens have served as targets for the development of cancer vaccines and engineered T-cell-based therapeutics. Therapeutic approaches including peptide-based CT antigen vaccines, engineered T cells, and lentiviral vector-based vaccines have been the foundation of multiple clinical trials and shown clinical promise [21,22,23,24,25,26]. Examples of specific trials in the context of HNSCC will be discussed in a later section.

### 2.3. Regulation of CT Gene Expression

Epigenetic mechanisms, particularly DNA demethylation, play a prominent role in the aberrant expression of CT genes in cancers [27]. For example, demethylation of CpG sites in the *MAGE-A1* promoter is correlated with its expression in cancers [28,29]. Treatment with the demethylating agent 5′-aza-2-deoxycytidine promotes the expression of a spectrum of CT genes in different cellular backgrounds [28,29,30]. DNA methyltransferases DNMT1 and DNMT3b appear to play a role in silencing, as loss of both leads to de-repression of the CT genes *TPTE*, *BRDT*, and *SYCP1* in colon cancer cell lines [30]. Acetylation may also play a role in expression. While treatment of cancer cell lines with histone deacetylase (HDAC) inhibitor trichostatin A led to minimal effects on MAGE-A gene expression, treatment with both trichostatin A and DNA methylase inhibitor 5-aza-CdR led to synergistic expression increases over 5-aza-CdR alone [31]. CT gene expression can also be modulated by transcription factors or regulators such as Sp1, p53, or the Brother of the Regulator of Imprinted Sites (BORIS), a paralog of the 11-zinc-finger gene regulator CTCF [27,32,33,34,35]. Such findings may be leveraged therapeutically to amplify CT antigen expression and enhance the efficacy of immunotherapies.

### 2.4. Hallmarks of Cancer Co-Opted by CT Antigens

Despite profound interest in the promise of CT antigens as immunotherapy targets, a fundamental question in the field is whether CT antigens play a mechanistic role as drivers of carcinogenesis or whether their expression is merely a bystander consequence of oncogenic transcriptional reprogramming. A burgeoning body of evidence suggests active roles for many of these proteins in oncogenic progression. Hanahan and Weinberg defined the “Hallmarks of Cancer”, a framework for understanding acquired cellular capabilities that enable carcinogenic initiation and progression [36]. While the oncogenic mechanisms of CT antigens have been reviewed elsewhere [9,10,27,37], the following provides examples of how CT antigens are involved in regulating hallmarks of cancer.

#### 2.4.1. Sustaining Proliferative Signaling, Resisting Cell Death, Evading Growth Suppressors

In a multidimensional siRNA-based functional screen, 140 CT antigens were independently depleted in 11 cancer cell lines. This was followed by an assessment of viability, apoptosis, and proliferation. Through this screen and subsequent validation, NY-ESO-1, FTHL17, and SPATA19 were identified as required for tumor cell proliferation and COX6B2 and CALR3 as essential for survival. Other factors validated to affect apoptosis, viability, or proliferation included MAGEA8, MAGEA2, SSX1, CTAG1B, IGSF11, CSAG1, CSAG3, CCDC110, ZNF165, and FATE1 [38].

Elsewhere in the literature, numerous CT antigens, including but not limited to CT45A1, PIWIL2, COX6B2, SPANX, DDX43, MAGE family members, CAGE, and SPAG6, have been implicated in cell viability, proliferation, clonogenic growth, and/or anchorage-independent colony growth [39,40,41,42,43,44,45,46,47,48,49]. CT antigens have been shown to inhibit apoptosis and promote resistance to chemotherapeutics. Multiple CT antigens share mechanisms that converge on the p53 tumor suppressor, a transcription factor that engages cytostatic or cytotoxic responses to various cellular insults and is the most frequently mutated gene in human cancers [50]. For example, MAGEA2, which promotes cancer cell resistance to etoposide treatment, interacts with the DNA binding domain of p53, thereby inhibiting p53-mediated activation of target gene expression [51,52]. MAGEA2 also impairs p53 acetylation, a stabilizing post-translational modification that enhances p53′s transcriptional activity [52,53]. CSAG2 inhibits p53 by promoting its deacetylation by SIRT1, which may mediate resistance to genotoxic stressors such as doxorubicin or H_2_O_2_ [43]. CAGE, whose overexpression confers resistance to chemotherapeutic agents such as taxol, also negatively regulates p53 expression [54].

#### 2.4.2. Activating Invasion and Metastasis and Inducing Angiogenesis

CT45A1 overexpression in osteosarcoma cells significantly increases the number of lung metastases in an in vivo metastasis assay [39]. Cell invasion and migration, two key cellular capabilities integral to the metastatic cascade [55], are also enhanced when CT45A1 is ectopically expressed in cells [39]. OIP5 is required for efficient in vitro invasion and migration of glioblastoma cells [49]. CAGE has been shown to promote both the metastatic and angiogenic potential of cancer cells [56].

#### 2.4.3. Deregulating Cellular Energetics

In cancers, energy metabolism is reprogrammed towards an increased reliance on glycolysis even in the presence of sufficient oxygen. This switch to aerobic glycolysis is hypothesized to funnel glycolytic intermediates into various biosynthetic pathways that support the cells’ proliferative drive, such as generating nucleosides and amino acids [36]. The CT antigens semenogelin 1 and 2 (SEMG1 and SEMG2) interact with and increase the levels and activity of metabolic enzymes: both affect pyruvate kinase M2 (PKM2), and SEMG1 also affects lactate dehydrogenase A (LDHA) [57]. PKM2 catalyzes the last step in glycolysis [58] while LDHA catalyzes the conversion of pyruvate to lactate and contributes to aerobic glycolysis [59], and both have been implicated as pro-tumorigenic proteins [58,59]. Overexpressing SEMG1 and SEMG2 also increases mitochondrial membrane potential (MMP) as well as both glycolysis and oxygen consumption rate [57]. Sperm are energetically demanding cells, requiring increased ATP for motility, and thus possess tissue-specific protein isoforms for both glycolysis and oxidative phosphorylation (OXPHOS) that allow them to meet these enhanced demands. COX6B2, a testis-specific subunit of cytochrome c oxidase (complex IV), enhances mitochondrial OXPHOS in tumor cells, which in turn promotes proliferation. COX6B2 is also induced by hypoxia and promotes cell proliferation in the face of hypoxic conditions [42]. MAGEA6 expression increases cellular growth compared to control cells following glycolytic inhibition with 2-deoxy-D-glucose (2-DG) [60]. 2-DG-treated MAGEA6-expressing cells enhance the synthesis of fatty acids. Concomitant treatment with etomoxir, a fatty acid oxidation inhibitor [61], attenuates the MAGEA6-induced growth advantage under conditions of 2-DG challenge, suggesting that MAGEA6 drives cell growth through fuel switching to fatty acid oxidation [60].

#### 2.4.4. Genome Instability

In triple-negative breast cancers (TNBCs), HORMAD1 expression can increase structural chromosomal abnormalities and chromosomal instability, in particular allelic-imbalanced copy number aberrations (AiCNA). HORMAD1 represses homologous recombination (HR) and inhibits the repair of spontaneously generated double-stranded DNA breaks (DSB). Nonhomologous end joining (NHEJ), a DSB repair process often upregulated in the context of HR deficiency, is upregulated by HORMAD1 overexpression, which can facilitate AiCNA formation. HR-deficient cancer cells can exhibit enhanced sensitivity to platinum-based chemotherapeutics and poly (ADP-ribose)-polymerase (PARP) inhibitors. Accordingly, HORMAD1-overexpressing TNBCs exhibit enhanced sensitivity to cisplatin and PARP inhibitors [62].

Furthermore, HORMAD1 overexpression abrogates DNA mismatch repair in ovarian and alveolar adenocarcinoma as well breast ductal carcinoma cell lines. This perturbation depends on interactions between HORMAD1 and the MCM8-MCM9 complex. High HORMAD1 overexpression is associated with increased tumor burden and neoantigen counts in breast invasive carcinoma, head and neck squamous cell carcinoma, lung adenocarcinoma, and thymoma in an analysis of genomes deposited in The Cancer Genome Atlas (TCGA). TCGA analysis has also revealed associations between HORMAD1 expression and genomic instability features including copy number alteration and loss of heterozygosity [63].

In summary, CT antigens have been reported to promote almost all hallmarks of cancer and regulate known oncogenic and tumor-suppressive signaling modules. This growing body of evidence suggests that the expression of CT antigens is more than an artifact and may drive carcinogenic processes.

## 3. HNSCC and CT Antigens

The expression of CT genes in HNSCC has been noted in the literature for over 20 years; some of the earliest reports include the detection of MAGEA3 [64], GAGE-1, and GAGE-2 [15], and LAGE-1 [65] expression in tumor tissues. Since then, studies have noted the expression of other CT antigens in HNSCC and analyzed correlations between expression and prognosis. These findings, which underscore the relevance and potential of CT antigens to serve as therapeutic targets and clinical course biomarkers in HNSCC, are summarized in Table 1.

Multiple clinical trials leveraging CT antigens in the context of HNSCC have been completed or are ongoing. A phase II randomized controlled clinical trial was completed, examining the safety and efficacy of MAGEA3 vaccine GL-0817 in combination with adjuvants for the prevention of oral squamous cell carcinoma recurrence in high-risk patients (NCT02873819) [121,122]. A phase I trial was completed examining the safety, kinetics, and clinical effect of T lymphocytes transduced with a MAGEA4-specific T-cell receptor gene in patients with unresectable, treatment-refractory solid tumors, including HNSCC (NCT02096614) [123]. Phase I trials have been completed investigating engineered T-cell receptor T lymphocyte therapy targeting NY-ESO-1 in patients with treatment-refractory solid tumors including HNSCC (NCT03159585, NCT02366546) [121,124,125]. The results for these trials have not yet been released. A nonrandomized phase II clinical trial was conducted in which advanced-stage HNSCC patients resistant to standard therapy were vaccinated with peptides derived from CT antigens Ly6K, CDCA1, and IMP3. Vaccinated patients exhibited significantly longer overall survival times than those receiving the best supportive care. Not all patients in the vaccination arm exhibited a cytotoxic T-lymphocyte (CTL) response, although those with a CTL response to LY6K or CDCA1 exhibited significantly longer overall survival compared to non-responders: 8.1 vs. 1.4 months and 11.3 vs. 4.6 months, respectively. After dividing vaccinated patients into groups based on the number of antigens to which they mounted a CTL response, the overall survival was longer for those who responded to more antigens, with a 19.5-month mean survival time in patients with CTL responses to all three antigens [126]. CT antigens thus hold potential as immunotherapeutic targets in HNSCC.

## 4. Contribution of CT Antigens to HPV-Associated HNSCCs

### 4.1. HPV Molecular Biology and Mechanisms of Carcinogenesis

HPVs are non-enveloped double-stranded DNA viruses with an approximately 8000 base-pair genome. They are divided into five genera: alpha, beta, gamma, mu, and nu. The alpha genus HPVs, which primarily infect mucosal epithelia, are further subdivided into the high-risk and low-risk types. Nearly all cases of cervical cancer and a large proportion of oropharyngeal and other anogenital cancers are caused by high-risk alpha HPVs. The low-risk alpha HPVs are not etiological agents in cancer—rather, they are associated with benign wart pathologies. The beta and gamma genus HPVs primarily infect cutaneous epithelia. Beta genus HPVs are the etiological agents of cutaneous squamous cell carcinoma (cSCC) in patients with the rare genetic disorder Epidermodysplasia Verruciformis and may contribute to cSCC in immunocompromised patients. E6 and E7 are the primary viral oncoproteins [127]. A vast body of literature has accumulated on the biological activities and oncogenic mechanisms of these proteins, which are reviewed elsewhere [127,128,129,130].

HPV-positive HNSCCs represent a distinct clinical entity that has a favorable prognosis compared to HPV-negative HNSCCs [131]. The enhanced survival rate is thought to be due to higher chemotherapy and radiotherapy sensitivity, although the underlying mechanisms are not completely understood [132]. The following section will explore the relevance of CT antigens to HPV-associated HNSCC.

### 4.2. CT Antigen Expression in HPV-Positive HNSCCs

The literature is sparse regarding the role of CT antigens in HPV-positive cancers. There is evidence to suggest that immune responses against CT antigens are elicited in some HPV-positive HNSCC patients and may serve as prognostic biomarkers. One study examining antibody responses to 16 CT antigens in HNSCC patients identified a subset of HPV-positive patients who exhibited responses, although there was considerable heterogeneity in the target signatures [133]. A related study examined prognostic trends in HNSCC patient antibody responses to 16 CT antigens, stratified by HPV status. Among HPV-positive patients, antibody response to IMP-1 was associated with significantly shorter overall survival: mean 109.3 months for IMP-1 response positive patients compared to 41.2 months for negative response patients. Indeed, HPV-positive patients with an IMP-1 antibody response had a prognosis that was not significantly different compared to HPV-negative patients [8]. Although HPV-positive HNSCC patients typically exhibit an improved prognosis compared to HPV-negative patients [131], there is still a subset of patients who are considered poor responders to treatment [134]. The differential survival outcomes among HPV-positive HNSCC patients based on antibody response to IMP-1 suggest that patients could be stratified by CT antigen signature to predict prognosis and identify such poor responders. Given the considerable morbidity associated with current HNSCC treatments, there is great interest in chemotherapy and radiotherapy dose de-escalation [135]. However, some de-escalation trials have shown detrimental survival outcomes with therapy reduction [135], which may be due in part to the inclusion of poor therapy responders. CT antigen signatures may serve as biomarkers to identify subsets of HPV-positive patients for which radiotherapy dose de-escalation may be safely pursued. The above reported studies examined only 16 CT antigens, a fraction of the documented repertoire. Additional studies assessing a broader range of CT antigens are warranted to identify signatures that can serve as biomarkers for prognosis and identifying candidate patients for dose de-escalation.

There is evidence that HPV-positive cervical cancer patients have benefited from lymphocytic targeting of CT antigens. One study examined the tumor antigen landscape of two patients with HPV positive metastatic cervical carcinoma—one HPV16+ squamous cell carcinoma and one HPV18+ adenocarcinoma—who achieved complete cancer regression following adoptive transfer of tumor-infiltrating lymphocytes (TIL) [136]. For the HPV18+ patient, analysis of T-cell antigens revealed reactivity to the CT antigen Kita-kyushu lung cancer antigen 1 (KK-LC-1) in addition to HPV16 E7. T-cells targeting KK-LC-1 represented 67% of the infused TILs, while those targeting HPV E7 represented only 14%. Tumor antigen-specific T-cell clonotypes were tracked in peripheral blood mononuclear cells (PBMCs) longitudinally at multiple time points following remission. KK-LC-1 clonotypes were more prevalent than the HPV E7 clonotypes by ≥10-fold, a trend that persisted through time [136]. The high proportion of KK-LC-1-targeting T cells among TILs and circulating PBMCs during tumor regression suggests that CT antigen targeting can play a role in clinical responses to immunotherapy in HPV+ cancers. While HPV oncoproteins E6 and E7 are considered attractive immunotherapeutic targets, targeting them may be insufficient to produce meaningful clinical outcomes. In a phase I/II trial testing engineered T cells expressing T-cell receptor against HPV16 E6, only 2/12 treated patients exhibited tumor responses [137]. Understanding the landscape of CT antigen expression induced by HPVs may help define synergistic targets to heighten immunotherapeutic responses. 

Multiple studies comparing gene expression differences between HPV-positive versus HPV-negative HNSCC have found multiple CT genes to be differentially regulated. CT genes that are significantly upregulated in HPV-positive versus negative HNSCCs are listed in Table 2. The following section elaborates on the physiological functions and potential oncogenic mechanisms of select upregulated CT genes identified in at least two independent studies.

### 4.3. SYCP2

The synaptonemal complex protein 2 (SYCP2) protein is a component of the synaptonemal complex, which joins homologous chromosomes prior to meiotic recombination [145]. The synaptonemal complex consists of two axial/lateral elements, a central element, and transverse filaments [146]. During the first stage of meiotic prophase I, called leptotene, chromosomes condense, and the sister chromatids organize along the axial elements. Meiotic DNA double-stranded breaks (DSBs) are formed and sequence matching on the homologous chromosome aligns the axial elements. During the next stage, zygotene, transverse filaments connect the axial elements, continuing until the length of the chromosome is joined by the synaptonemal complex. This state is called synapsis and is achieved by the beginning of the pachytene stage. The axial elements are incorporated into the synaptonemal complex as lateral elements. The pachytene stage includes the formation and resolution of the double Holliday Junction into crossovers. During diplotene, the synaptonemal complex disassembles and homologous chromosomes separate except at chiasmata [147,148]. SYCP2 is required for the formation of axial elements as well as synapsis during male meiosis [148,149,150]. In mice, *Sycp*2 knockout leads to reduced female fertility, male sterility and meiotic arrest, and spermatocyte apoptosis [148]. In zebrafish spermatocytes, in addition to its role in synaptonemal complex assembly, SYCP2 is also important for homologous pairing and meiotic double-stranded break formation [151]. While few studies have focused on the role of SYCP2 in cancer, others provide further evidence that SYCP2 expression is characteristic of HPV-associated cancers. *SYCP2* was found to be one of the six most differentially expressed genes in HPV-positive cervical cancer compared to cervical epithelium control tissues and exhibits increasing expression levels in the progression from normal tissue to pre-cancerous lesions to cervical cancer [152,153]. SYCP2 expression was detected in HPV16 positive but not negative keratinocyte lines and expression was promoted synergistically by HPV16 E6 and E7 [139]. SYCP*2* expression has been noted in pre-malignant HPV-positive oropharyngeal tissue, and high SYCP2 expression in HNSCC has been associated with improved disease-free survival [142]. Multiple synaptonemal complex components, including SYCP1, SYCP2, SYCP3, SYCE1, and SYCE2, are expressed in cancers [146]. Expression of synaptonemal complex axial/lateral element SYCP3 [146] in somatic cells impairs the RAD51-mediated homologous recombination pathway, enhances sensitivity to DNA damaging agents or poly(ADP-ribose) polymerase (PARP) inhibition, and promotes chromosomal instability. SYCP3 interacts with BRCA2 and can inhibit its function in homologous recombination [154]. Given that HPV16 E6 and E7 can induce DNA damage [155] and promote genomic instability [156], it is conceivable to hypothesize that SYCP2 upregulation might promote this hallmark of cancer.

### 4.4. ZCWPW1

Zinc finger CW-type and PWWP domain containing 1 (ZCWPW1) is a reader of histone H3 trimethylation marks on lysine 4 and/or lysine 36 (H3K4me3 and H3K36me, respectively) [157,158]. In meiosis, H3K4me3 and H3K36me3 mark meiotic DSBs and are written by the methyltransferase PR domain zinc finger protein 9 (PRDM9) [157,159,160,161]. ZCWPW1 binds to these dual PRDM9-dependent histone methylation marks at meiotic recombination DSB hotspots [157,158,162]. *Zcwpw1* loss in male mice leads to azoospermia [157] and impairs meiotic prophase I processes including synapsis, meiotic recombination, and meiotic DSB repair [157,162,163]. The precise mechanism for how ZCWPW1 might facilitate DSB repair is unknown, although one hypothesis is that it nucleates repair machinery [158]. Although ZCWPW1 binds with higher affinity to dual H3K4me3/H3K36me3 marks, it can also bind to each mark individually [157]. Independently of PRDM9, ZCWPW1 can bind to CpG dinucleotides and interact with Alu repeats in a CpG-dependent manner - it has a greater affinity for methylated CpGs, although it can also to bind non-methylated CpGs [162]. While no studies have investigated a potential mechanistic link between ZCWPW1 and cancer, H3K4 and H3K36 readers play a role in diverse cancers [164,165], as do methyl-CpG binding proteins [166].

### 4.5. TAF7L

TATA-binding protein-associated factor 7L (TAF7L) is an X-linked paralog of the transcription factor IID (TFIID) subunit TAF7 that is predominantly restricted to germ cells in the testis [167]. TFIID is a critical factor for the initiation of RNA polymerase II-mediated gene transcription. It consists of the TATA-binding protein (TBP), which binds to the TATA element present in many promoters, in addition to 13-14 TBP-associated factors (TAFs). TAF7L is expressed at multiple stages in the germ cell differentiation process, including in spermatogonia, spermatocytes, and haploid round spermatids. TAF7L interacts with TBP and TFIID subunit TAF1 [167]. Loss of *Taf7l* in male mice (*Taf7l* ^-/Y^) leads to smaller litters, sperm tail structural defects, and decreased sperm motility [168], and continued back-crossing of *Taf7l* ^-/Y^ mice can lead to sterility [169]. *Taf7l* ^-/Y^ testes exhibit pronounced gene expression remodeling, with notable downregulation of genes involved in spermatogenesis, sperm motility, and metabolism [168,169]. Of note, expression of Sex comb-like with four MBT domains 2 (SFMBT2), a Polycomb group (PcG) protein that may have an oncogenic role [170], was downregulated with *Taf7l* ablation [168]. While TAF7L was initially thought to be testis-specific, studies later revealed its expression in adipocytes and suggested a role in adipocyte differentiation [171]. TAF7L can also function as a molecular switch specifying brown fat or muscle cell fate, with TAF7L expression favoring brown adipose tissue formation [172]. Aberrant TAF7L expression might lead to transcriptional reprogramming that can promote cancer progression.

### 4.6. STAG3

Stromal antigen 3 (STAG3) is a germline and meiosis I-specific subunit of cohesin [173]. Cohesin is a ring-shaped four-subunit complex that mediates sister chromatid cohesion, a function that is vital for chromosome segregation and DNA repair [174]. Cohesins comprise two structural maintenance of chromosomes (SMC) proteins, the kleisin subunit, and a stromal antigen (STAG) [174]. In somatic cells, the SMC proteins are SMC1 and SMC3, the kleisin subunit is RAD21, and the stromal antigen is STAG1 or STAG2 [174]. In the germline, the meiosis-specific cohesin proteins are the SMC protein SMC1β, kleisin REC8 or RAD21L, and the stromal antigen STAG3 [174,175]. STAG3 loss is associated with sterility, disrupted synaptonemal complex assembly and synapsis, impaired centromeric and telomeric sister chromatid cohesion, and dysfunctional meiotic recombination [176,177,178,179]. Loss of STAG3 also leads to impaired repair of programmed double-stranded breaks (DSBs) and defective DNA damage response [178,179]. Ataxia-telangiectasia and RAD3-related (ATR), a serine/threonine kinase, is typically activated by single-stranded DNA (ssDNA) and triggers downstream responses including cell cycle arrest, DNA repair, fork stabilization, and apoptosis in somatic cells [180]. In meiosis, during zygotene, ATR and ATR interacting protein (ATRIP) activate the DNA damage response to signal the presence of recombination intermediates. ATR typically localizes to unsynapsed chromosome regions during zygonema and dissociates following synapsis. *Stag3* mutant mice exhibit aberrant ATR and ATRIP localization [179]. STAG3 has been shown to interact with PRDM9, which marks recombination hotspots via its histone methyltransferase activity, and to promote meiotic programmed DSBs at both PRDM9-dependent and independent hotspots [181]. Mechanistically, STAG3 facilitates localization of DSB-promoting proteins HORMAD1, IHO1, and MEI4 to the chromosome axis and mediates DSB-forming activities of SPO11 [181].

STAG3 expression is silenced in somatic cells by E2F6, a transcriptional repressor that is a component of Polycomb repressive complexes (PRC) [182]. MAX Gene-Associated (MGA), a component of non-canonical PRC1.6, also mediates the repression of STAG3 and other meiotic genes [183]. Given that HPV16 E7 interacts with E2F6, inhibits its transcription repressive functions, and attenuates the Polycomb group (PcG)-mediated formation of heterochromatin-associated nuclear foci [184], one might hypothesize that HPV promotes aberrant STAG3 expression through abrogation of E2F6 repression.

Although the physiological role of STAG3 is actively being characterized, there are fewer studies on its role in carcinogenesis. Loss of STAG3 in melanoma leads to BRAF inhibitor resistance [185]. However, STAG3 overexpression is also a common event in various cancers [186]. In HNSCCs not stratified by HPV status, STAG3 expression is associated with improved overall survival and progression-free interval [143]. Conversely, high STAG3 expression is associated with poor prognosis, metastasis, and disease recurrence in colorectal cancer patients [187]. In colorectal cancer cell lines, STAG3 downregulation enhances sensitivity to chemotherapeutics and impairs DNA damage repair [187]. Cohesin complex components are frequently mutated in cancers and mitotic stromal antigen 2 (*STAG2*) is the second most commonly mutated gene in Ewing sarcoma [188,189]. In line with their role in chromosome segregation, somatic mutations in cohesin subunit genes have been reported to promote chromosomal instability in cancers. This is an “enabling hallmark” of cancer that is characterized by higher rates of chromosome mis-segregation during mitosis, leading to aneuploidy as well as translocations and loss of heterozygosity, among other defects [188,190,191,192]. However, cohesin subunit mutations have also been reported in chromosomally stable tumors without correlation between mutation and aneuploidy, suggesting that cohesin subunit mutations may drive oncogenesis through alternative mechanisms unrelated to chromosomal instability [188,193,194]. While these studies suggest a tumor-suppressive role for cohesin subunits, aberrant cohesin overexpression is observed in certain cancers and there is evidence that this may activate oncogenic transcription [195]. Given its meiotic activities, one might hypothesize STAG3 to interplay with the DNA damage response and play a role in promoting genomic instability. Further investigations are warranted to understand how STAG3 affects carcinogenesis in HPV-associated cancers.

## 5. Conclusions

Since their discovery, CT antigens have ignited interest as promising targets for cancer immunotherapies. Many of them are expressed in HNSCC and the possibility of their clinical translation in the context of engineered T cells and cancer vaccines is already being investigated in clinical trials. Although the viral antigens within HPV-positive cancers are immunogenic and numerous clinical trials testing immunotherapies targeting HPV16 E6/E7 are underway, the response rates have been disappointing. For example, the ISA 101 trial that tested peptide vaccines targeting HPV16 E6/E7 demonstrated a 33% response rate [121]. As CT antigen-targeted vaccines have a demonstrated survival benefit in the context of both HSNCC and non-HNSCC cancers [21,22,126], targeting both upregulated CT antigens and viral antigens may be one strategy to improve immunotherapeutic responses. HPV-positive HNSCC represents a distinct clinical entity with improved prognosis compared to HPV-negative cancers [131]. Despite this improved prognosis, the current non-surgical standard-of-care treatment—high-dose cisplatin concurrent with radiotherapy—is associated with considerable morbidity. As such, there is an impetus to de-escalate treatment in HPV-positive patients via reducing radiation dose and/or type and dose of chemotherapy. Given that antibody response to CT antigens such as IMP-1 can stratify HPV-positive HNSCC based on prognosis, it may be possible to use CT antigen signatures as biomarkers for chemoradiotherapy response to identify those most likely to benefit from de-escalation. The mechanism of the enhanced therapeutic sensitivity and improved prognosis associated with HPV-positive HNSCC is not fully understood [132]. Considering that some CT antigens upregulated in HPV-positive cancers may promote genomic instability [37] and that high expression of *SYCP2* or *STAG3* correlates with improved survival in HSNCC [142,143], it is conceivable that aberrant CT antigen expression may contribute to tumor chemo- and radiosensitivity. Although published correlations between CT antigen expression and prognosis in HPV-positive HNSCC are limited, because there is precedent that CT antigens can promote nearly all hallmarks of cancer [36] but many, including those upregulated in HPV-positive HNSCC, do not have formally reported oncogenic cellular functions, studying how CT antigens contribute to HPV-mediated oncogenesis is an intriguing field of study that provides a unique opportunity to uncover novel oncogenic mechanisms and reveal potential new drug targets or collateral sensitivities.

## Figures and Tables

**Table 1:** Expression and clinical correlates of CT antigens in HNSCC.

Expressed CT Gene/Antigen	Oncogenic Functions	Prognostic Associations in HNSCC	References Reporting Expression in HNSCC Tumors
MAGEA1	Proliferation, invasion, migration [46]	Associated with tumor regional recurrence, worse overall survival among HPV-negative patients and all patients not stratified by HPV status [8,66]	[8,66,67,68,69,70,71,72]
MAGEA2	Proliferation, suppress cell cycle arrest through p53 [73]		[69]
MAGEA3	Proliferation, migration, invasion [74,75,76]	Associated with tumor regional recurrence, worse overall survival [8,66]	[8,64,66,69,70,77]
MAGEA3/6	Proliferation, migration, invasion, anchorage-independent growth [78,79,80]	Associated with improved disease-free survival [71]	[71]
MAGEA4	Activate trans-lesion synthesis (genomic instability), inhibit apoptosis, inhibit growth arrest [81,82]	Associated with worse overall survival among HPV-negative patients and all patients not stratified by HPV status [8]	[8,66,69,71,72,82,83,84,85,86]
MAGEA6	Inhibit cell death [87]		[69]
MAGEA9	Proliferation, migration, chemoresistance [88]	Associated with worse overall survival [8]	[8,71]
MAGEA10			[72]
MAGEA11	Resistance to epidermal growth factor receptor inhibitors [89]	Associated with worse 5-year overall survival rate [90]	[89,90,91]
MAGEA12	Migration, invasion [92]		[71,72]
MAGEB2	Proliferation [78,93,94]		[71]
MAGEB6			[71]
MAGEC1	Inhibit apoptosis [48]		[66,68,71,77,83,95]
MAGEC2	Proliferation, amoeboid migration, metastasis [78,96,97]		[66,68,71,77,95,98]
NY-ESO-1		Associated with worse overall survival [68]	[65,66,68,70,72,77,85,95,98]
SSX	In vivo tumor growth, invasion, migration [99,100]	Associated with worse overall survival [8]	[8,77,98]
IMP1	Invasion, promotion of stemness [101]	Associated with worse overall survival among HPV-positive patients and among all patients not stratified by HPV status [8]	[8]
SAGE			[77,85,95]
BAGE			[77]
GAGE	Anti-apoptotic activity, radioresistance, chemoresistance [102]	Associated with lymph node metastases [91]	[15,72,77,91,98,103]
CRISP2			[71]
PRAME	Binds to retinoic acid receptor (RAR) and inhibits its transcriptional activation. Inhibits differentiation, apoptosis, arrest of proliferation typically induced by retinoic acid. [104,105]		[71,72,106]
NY-TLU-57			[77]
SPANX	Proliferation [44]		[71]
CXORF48			[71]
HOM-TES-85			[77]
SYCP1			[77]
CT45	Stemness, chemoresistance [107]		[95]
	Chemosensitivity [108]		
NXF2			[95]
XAGE1		Associated with lymph node metastases [91]	[91]
CTAGE			[109]
SP17	In vivo tumor growth, chemoresistance, migration [110,111]		[112]
BRDT	Proliferation, migration, inhibition of apoptosis [113,114]		[115]
ACTL8	Proliferation, invasion, migration [116,117]	Associated with worse prognosis [117]	[117]
PLAC1	Proliferation, migration, invasion [118,119]		[120]

**Table 2:** CT genes significantly upregulated in HPV-positive HNSCC compared to HPV-negative HNSCC. Wang et. al., 2016 [19] was used to define the CT gene reference list.

CT Gene	Reference
*SYCP2*	[134,138,139,140,141,142]
*STAG3*	[134,139,143]
*TAF7L*	[134,138,139]
*YBX2*	[134,138]
*RIBC2*	[134,143]
*ZCWPW1*	[134,143]
*POU4F1*	[134,144]
*DDX43*	[144]
*LDHC*	[134]
*TCP11*	[134]
*FKBP6*	[134]
*SOX30*	[134]
*SMC1B*	[134]
*DDX25*	[134]
*YPEL1*	[134]
*KIF15*	[134]
*CENPH*	[134]
*C19orf57*	[134]
*BCL2L14*	[134]
*SHCBP1L*	[134]
*ZNF541*	[134]
*IZUMO4*	[134]
*ZPBP2*	[134]
*CNTD1*	[134]
*RAD9B*	[134]
*CCDC155*	[134]
*SYCE2*	[134]
*PRR19*	[134]
*KIF24*	[134]
